# Age-Dependent Changes in the Propofol-Induced Electroencephalogram in Children With Autism Spectrum Disorder

**DOI:** 10.3389/fnsys.2018.00023

**Published:** 2018-06-22

**Authors:** Elisa C. Walsh, Johanna M. Lee, Kristina Terzakis, David W. Zhou, Sara Burns, Timothy M. Buie, Paul G. Firth, Erik S. Shank, Timothy T. Houle, Emery N. Brown, Patrick L. Purdon

**Affiliations:** ^1^Department of Anesthesia, Critical Care and Pain Medicine, Massachusetts General Hospital, Boston, MA, United States; ^2^Harvard Medical School, Boston, MA, United States; ^3^Harvard Medical School/Massachusetts Institute of Technology, Division of Health Sciences and Technology, Cambridge, MA, United States; ^4^College of Nursing, Villanova University, Villanova, PA, United States; ^5^Lurie Center for Autism, Mass General Hospital for Children, Boston, MA, United States; ^6^Department of Gastroenterology, Mass General Hospital for Children, Boston, MA, United States; ^7^Department of Brain and Cognitive Science, Massachusetts Institute of Technology, Cambridge, MA, United States; ^8^Institute for Medical Engineering and Sciences, Massachusetts Institute of Technology, Cambridge, MA, United States

**Keywords:** autism spectrum disorder (ASD), electroencephalography (EEG), propofol, gamma aminobutyric acid (GABA), general anesthesia

## Abstract

Patients with autism spectrum disorder (ASD) often require sedation or general anesthesia. ASD is thought to arise from deficits in GABAergic signaling leading to abnormal neurodevelopment. We sought to investigate differences in how ASD patients respond to the GABAergic drug propofol by comparing the propofol-induced electroencephalogram (EEG) of ASD and neurotypical (NT) patients. This investigation was a prospective observational study. Continuous 4-channel frontal EEG was recorded during routine anesthetic care of patients undergoing endoscopic procedures between July 1, 2014 and May 1, 2016. Study patients were defined as those with previously diagnosed ASD by DSM-V criteria, aged 2–30 years old. NT patients were defined as those lacking neurological or psychiatric abnormalities, aged 2–30 years old. The primary outcome was changes in propofol-induced alpha (8–13 Hz) and slow (0.1–1 Hz) oscillation power by age. A *post hoc* analysis was performed to characterize incidence of burst suppression during propofol anesthesia. The primary risk factor of interest was a prior diagnosis of ASD. Outcomes were compared between ASD and NT patients using Bayesian methods. Compared to NT patients, slow oscillation power was initially higher in ASD patients (17.05 vs. 14.20 dB at 2.33 years), but progressively declined with age (11.56 vs. 13.95 dB at 22.5 years). Frontal alpha power was initially lower in ASD patients (17.65 vs. 18.86 dB at 5.42 years) and continued to decline with age (6.37 vs. 11.89 dB at 22.5 years). The incidence of burst suppression was significantly higher in ASD vs. NT patients (23.0% vs. 12.2%, *p* < 0.01) despite reduced total propofol dosing in ASD patients. Ultimately, we found that ASD patients respond differently to propofol compared to NT patients. A similar pattern of decreased alpha power and increased sensitivity to burst suppression develops in older NT adults; one interpretation of our data could be that ASD patients undergo a form of accelerated neuronal aging in adolescence. Our results suggest that investigations of the propofol-induced EEG in ASD patients may enable insights into the underlying differences in neural circuitry of ASD and yield safer practices for managing patients with ASD.

## Introduction

Autism spectrum disorder (ASD) is a neurodevelopmental disorder characterized by deficits in social interaction and restricted, repetitive patterns of behavior, interests and activities (American Psychiatric Association, 2013). Beyond its core features, ASD is a heterogeneous condition with significant variation in quality and severity of symptoms. Although once considered rare, ASD affected nearly 1 in 68 children in the U.S. in 2010, with a prevalence that continues to rise (Baio, 2014). The pathophysiology of ASD remains hotly debated, but a leading hypothesis in the field is that inhibitory γ-aminobutryic acid (GABA) signaling develops abnormally in the disorder.

Children with ASD are known to have an increased rate of overall hospital contact compared to those without ASD (Gurney et al., 2006; Petersen et al., 2006, 2009; Atladóttir et al., 2012). Due to frequent hypersensitivity to sensory stimuli, children with ASD often require sedation and general anesthesia for even minor procedures. Clinicians tend to give premedication with oral midazolam or ketamine to patients with ASD to prevent agitation. However, there are no guidelines at present on how to adjust anesthetic management in ASD patients, despite their neurodevelopmental differences.

One approach to guide anesthetic dosing is to employ electroencephalogram (EEG)-based brain monitoring. Studies in adult patients have shown that general anesthetic drugs induce stereotyped brain oscillations that can be readily measured using the EEG (Gibbs et al., 1937; Gugino et al., 2001; Feshchenko et al., 2004; Ching et al., 2010; Cimenser et al., 2011; Supp et al., 2011; Lewis et al., 2012; Lee et al., 2013; Purdon et al., 2013; Akeju et al., 2014a,b). During unconsciousness induced by propofol, a commonly used GABAergic anesthetic, the frontal EEG shows large-amplitude slow (0.1–1 Hz) and alpha oscillations (8–13 Hz; Lewis et al., 2012; Purdon et al., 2013, 2015b). The slow oscillation is thought to reflect periodic down states during which neurons are unable to fire, and recent studies have suggested that inhibitory networks play a key role in the generation of slow waves (Lewis et al., 2012; Funk et al., 2017; Zucca et al., 2017). The propofol-induced frontal alpha oscillation appears to be a GABA-dependent oscillations engaging the prefrontal cortex and thalamus (Ching et al., 2010; Flores et al., 2017). The anesthesia-induced EEG can also show burst suppression, a state of profound brain inactivation that is characterized by epochs of low amplitude activity or isoelectricity corresponding to electrocortical silence (“suppression”), interrupted by epochs of relative high amplitude activity (“bursts”; Kenny et al., 2014). The mechanism of burst suppression is not well characterized, but is hypothesized to reflect changes in potassium dynamics in the setting of altered neuronal metabolism (Ching et al., 2012).

The anesthesia-induced EEG has also been studied in pediatric patients. Previous studies show substantial age-related changes in the propofol–and sevoflurane-induced EEG in pediatric patients (Akeju et al., 2015; Cornelissen et al., 2015; Lee et al., 2017). Total EEG power, including slow and alpha oscillations, increases from 0 years old to 6 years old, peaks at approximately 6–8 years old, and declines with increasing age until age 21. These changes appear to mirror changes in cortical synaptic density during childhood and adolescence (Petanjek et al., 2011). Given that both propofol and sevoflurane act via allosteric modulation of GABA_A_ receptors, we hypothesized that the observed age-dependent changes in the EEG could reflect development of GABAergic inhibitory interneurons in the cerebral cortex and thalamus.

Given that GABA signaling is thought to be affected in ASD, we hypothesized that propofol-induced EEG oscillations in children with ASD might differ from those of neurotypically-developing children. To investigate this hypothesis, we performed a prospective observational study to compare the age-dependent propofol EEG dynamics in pediatric ASD and neurotypical (NT) patients.

## Materials and Methods

### Patient Selection and Data Collection

The Human Research Committee at the Massachusetts General Hospital (Boston, MA, USA) approved this prospective observational study. All data were collected in the pediatric endoscopy suite at the Massachusetts General Hospital. As no intervention was being performed, and since all physiologic measurements were non-invasive and part of routine clinical care, waiver of consent was granted for this study. We identified patients with previously diagnosed ASD using the DSM-V definition, which encompasses autism, Asperger’s syndrome, and pervasive developmental disorder not otherwise specified (PDD-NOS; Maenner et al., 2014). We defined NT patients as those lacking neurological or psychiatric abnormalities.

We recorded continuous 4-channel frontal EEG data using the SED Line brain function monitor (Masimo Corporation, Irvine, CA, USA) during routine endoscopies of 90 ASD patients and 157 NT patients aged 2–30 years between July 1, 2014 and May 1, 2016. All data were strictly observational; there was no experimental intervention, and the choices and dosing of any anesthetic drugs were made by the clinical anesthesiologists managing the patient. EEG data were recorded with a pre-amplifier bandwidth of 0.5–92 Hz, sampling rate of 250 Hz, and a 16-bit, 29 nV resolution, from an adhesive sensor array with electrodes positioned approximately at Fp1, Fp2, F7 and F8, with the ground electrode at Fpz and reference electrode at ~1 cm above Fpz.

**Figure 1:**
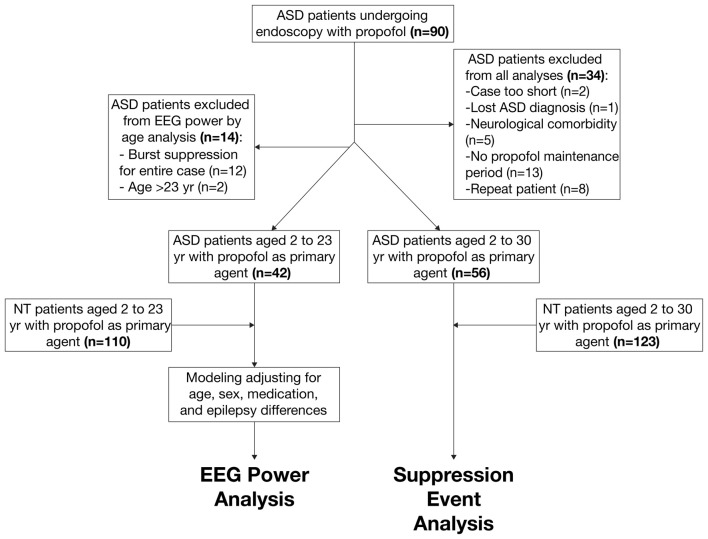
Schematic of patient selection procedure from autism spectrum disorder (ASD) and neurotypical (NT) cohorts.

The primary ASD (*n* = 42) and NT (*n* = 110) cohorts included patients between 2 years old and 23 years old who received propofol anesthesia with a sufficient period of maintenance anesthesia without noise, artifacts, or EEG suppression. We decided not to exclude for epilepsy in the ASD cohort, as epileptiform EEG patterns in ASD patients commonly exist even without clinically diagnosed epilepsy (Hashimoto et al., 2001). We selected these primary cohorts to analyze age-dependent EEG power in ASD and NT patients. The secondary ASD (*n* = 56) and NT (*n* = 123) cohorts included patients between 2 years old and 30 years old and did not exclude for the presence of suppressive events. We selected these secondary cohorts to analyze the incidence of burst suppression in ASD and NT populations. Figure 1 summarizes the procedure of patient selection for each cohort.

### Data Processing

We selected EEG data segments using information from both the electronic anesthesia record (Metavision, Dedham, MA, USA) and the EEG spectrogram. The concentrations of inhaled agents were automatically captured, and the concentrations of all other drugs were manually recorded in the electronic anesthesia record by the anesthesia providers. For each case, we identified a 120-second epoch with a stable propofol infusion rate *and* no other anesthetic drugs given for at least 5 min prior to the epoch. In cases with mask induction, we specified that epochs must occur ≥5 min after the discontinuation of inhaled anesthetic (sevoflurane ± N_2_O). Two of the authors (ECW, PLP) inspected the electronic medical records and EEG data for each subject to select data segments meeting the above criteria. These same authors carefully examined the data segments, visualizing both the EEG spectrogram as well as the unprocessed EEG waveform, to ensure that they were free of movement artifacts (e.g., patient re-positioning), environmental noise, and burst suppression.

### Spectral Analysis

We estimated the EEG power spectrum and spectrogram for each subject in the primary cohort using multitaper spectral analysis methods enabled by the Chronux toolbox (Percival and Walden, 1993). The EEG power spectrum quantifies the energy in the EEG signal at each frequency; the EEG spectrogram displays the EEG power spectrum as a function of time. We calculated the EEG power spectrum and spectrogram in each subject by averaging the spectrum or spectrogram, respectively, across the Fp1, Fp2, F7 and F8 channels. To illustrate the evolution of the spectrum with increasing age, we also calculated an age-varying spectrogram across each cohort by computing the median spectrum across patients in 0.5 year age bins, using an overlapping (0.5 year) sliding window spanning ±2 years at each age value from 2 years to 23 years.

### Burst Suppression Analysis

We scored each record from the secondary cohort for episodes of burst suppression or a prolonged suppression event occurring at any time during the case. Burst suppression was defined operationally as the presence of at least three consecutive suppression events within a 60-s period. A prolonged suppression event was defined operationally as the presence of a single suppression event lasting ≥10 s. Patients showing either burst suppression or a prolonged suppression event at any point during the case were graded with a “1,” whereas patients who did not show either event were graded with a “0.” Two separate lab members (ECW, PLP) reviewed all records and only those cases that were in agreement maintained a grade of “1.”

### Statistical Analysis

The primary outcomes were the propofol-induced EEG alpha (8–13 Hz) and slow (0.1–1 Hz) oscillation power as a function of age. The *post hoc* analysis was incidence of burst suppression. The primary risk factor of interest for both investigations was a prior diagnosis of ASD as defined by DSM-V criteria.

We presented continuous variables that were normally distributed as means (SD) and categorical variables as frequency counts (%). We compared preoperative characteristics between the ASD vs. NT patients using standardized mean differences (SMD). We considered an SMD greater than 0.2 (corresponding to a 15% non-overlap in the two distributions) be notable.

We performed a Bayesian regression analysis using Markov Chain Monte Carlo techniques. We based our results on 10,000 iterations after a burn-in period of 1000 iterations with thin set to 1. We used two separate models to examine alpha and slow waves. The primary linear regression models assume no prior probabilities and analyze differences in age-dependent power between ASD and NT groups while adjusting for sex, propofol infusion rate (mcg/kg/min), propofol bolus (mg/kg), midazolam (mg/kg), fentanyl (mg/kg), and comorbidity of epilepsy. We determined the optimal representation of age to be age^3^ according to the Bayes factor criteria, which is consistent with our prior analyses (Akeju et al., 2015; Lee et al., 2017). We reported the 50% posterior probability and corresponding 80% two-sided posterior density intervals of the slope. We did not perform an* a priori* statistical power calculation. We conducted all analyses using R statistical software (RStudio, version 3.2.2; R Foundation for Statistical Computing, Vienna, Austria).

To characterize the incidence of burst suppression in ASD and NT patients, we compared the proportion of ASD and NT patients displaying an episode of burst suppression using a Bayesian approach. We modeled this proportion as a beta distribution for each cohort wherein α = k + 1 where k = # cases with confirmed events and β = n + 1 where n = # of cases in a given cohort. We estimated the posterior distribution for each cohort to represent the probability (Pr) that patients experience an episode of burst suppression. We then estimated the posterior density for the difference between Pr_ASD_ and Pr_NT_ (ΔPr) wherein Pr_ASD_ > Pr_NT_ using a Markov Chain Monte Carlo approach. We chose a uniform prior distribution and used 10,000 Monte Carlo samples to compute the posterior density.

## Results

### Patient Population for Age-Dependent EEG Power Analysis

We included a total of 42 ASD patients and 110 NT patients aged 2–23 years in the age-dependent EEG power analysis. Patient baseline characteristics and anesthetic regimens are summarized in Table 1. Supplementary Figure S1 shows the age distribution for the ASD and NT groups, respectively, in histogram form.

**Table 1:** Baseline characteristics and anesthetic regimens in autism spectrum disorder (ASD) and neurotypical (NT) pediatric cohorts.

Parameter	ASD (*n* = 42)	NT (*n* = 110)	SMD
Age (years)	10.88 (5.25)	13.29 (5.27)	0.460
Male sex (%)	36 (85.7)	61 (55.5)	0.704
Propofol infusion rate (mcg/kg/min) during epoch	239.88 (32.69)	256.11 (37.15)	0.464
Propofol bolus (mg/kg) prior to epoch	1.34 (1.13)	2.03 (1.52)	0.514
Midazolam (mg/kg) prior to epoch	0.01 (0.03)	0.00 (0.01)	0.430
Fentanyl (mcg/kg) prior to epoch	0.71 (0.62)	0.74 (0.58)	0.046
Comorbid clinically diagnosed epilepsy (%)	4 (9.5)	0 (0.0)	0.459

*All values are means (SD). SD, standard deviation; SMD, standardized mean difference*.

There were notable differences in the characteristics of the ASD and NT cohorts. The mean age of ASD patients was less than that of NT patients (10.88 vs. 13.30 years, SMD = 0.460). Supplementary Figure S1 demonstrates a histogram of patients across each age for ASD and NT cohorts. ASD patients were also more likely to be male (85.7% vs. 55.5%, SMD = 0.704) and have comorbid epilepsy (9.5% vs. 0%, SMD = 0.459). Additionally, ASD patients received reduced propofol infusion rates (239.88 vs. 256.11 mcg/kg/min, SMD = 0.464) and propofol boluses (1.34 vs. 2.03 mg/kg, SMD = 0.514), but increased midazolam premedication (0.01 vs. 0.00 mg/kg, SMD = 0.430). There were no differences in fentanyl administration in ASD and NT patients (0.71 vs. 0.74 mg/kg, SMD = 0.046).

**Figure 2:**
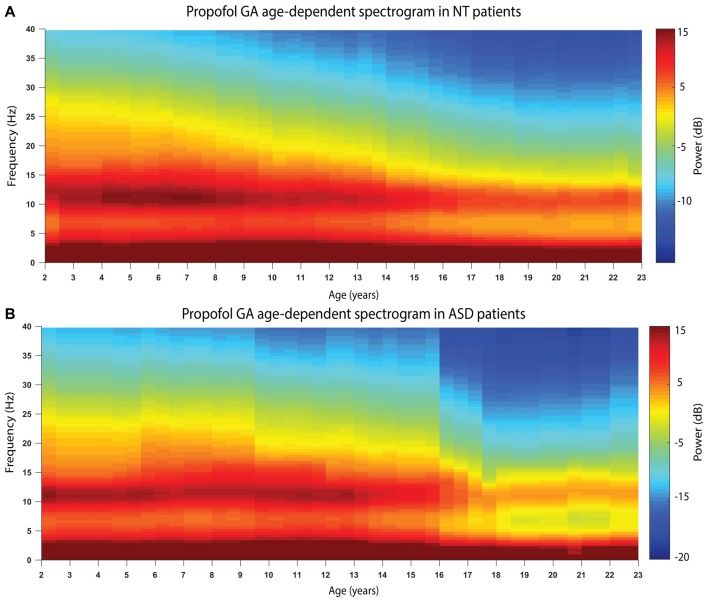
The age-varying spectrogram in **(A)** NT (*n* = 110) and **(B)** ASD (*n* = 42) patients during propofol-induced general anesthesia. Prominent slow-delta (0.1–4 Hz) and alpha (8–13 Hz) oscillations are present in all patients, which is the typical electroencephalogram (EEG) signature of propofol-induced general anesthesia. Total EEG power (0.1–40 Hz) declines with increasing age in both cohorts. Alpha (8–13 Hz) EEG oscillation power appears to decline more significantly in the ASD cohort compared to the NT cohort.

Figure 2 shows the age-varying spectrogram for ASD and NT patients aged 2–23 years. In both cohorts, total EEG power (0.1–40 Hz) as well as slow-delta (0.1–4 Hz) and alpha (8–13 Hz) power declines with increasing age. The decline of alpha oscillations appears to be exaggerated in ASD compared to NT patients.

### The Trajectory of Alpha (8–13 Hz) Power by Age Differs in ASD and NT Patients

To better assess the age-dependent changes in power in the propofol-induced EEG in ASD and NT patients, we used a Bayesian approach to investigate the relationship of EEG power and age in alpha and slow oscillations in two separate models.

**Figure 3:**
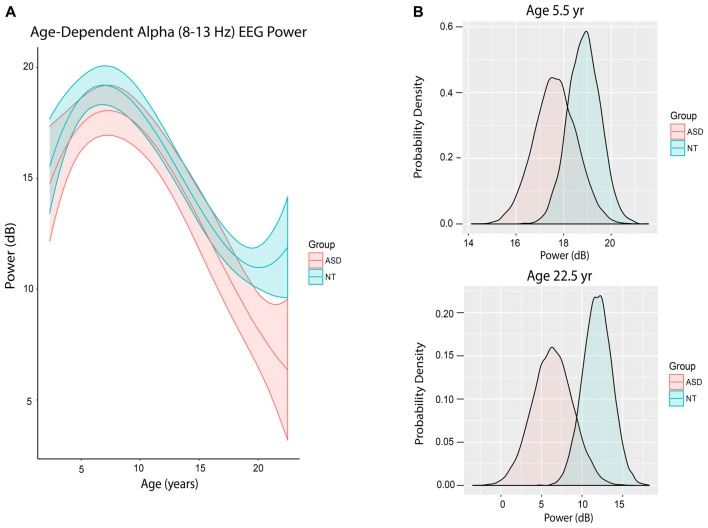
Alpha (8–13 Hz) EEG power evolves differently with age in ASD patients.** (A)** Alpha (8–13 Hz) power in the propofol-induced frontal EEG by age and ASD status. **(B)** Posterior distributions of alpha (8–13 Hz) EEG power (dB) in ASD vs. NT cohorts at 5.5 years and 22.5 years. At 5.5 years of age, ASD patients had lower alpha power with 56.5% certainty. At 22.5 years, ASD patients had lower alpha power with 80.2% certainty.

Figure 3A shows the trajectory of alpha power by age in ASD and NT patients after accounting for differences in baseline characteristics between the cohorts. ASD patients at mean age experienced a non-significant 0.47 dB reduction in alpha power compared to NT patients (80% credibility interval −1.82 to 0.82; Table 2).

**Table 2:** Posterior probabilities from primary model of alpha (8–13 Hz) power (dB) by using mean age.

Parameter	50%	80% HPD
Group = ASD	−0.469	(−1.820, 0.822)
Age	−0.077	(−0.109, −0.061)
Age 2	0.000	(−0.000, −0.000)
Age 3	0.000	(0.000, 0.000)
Gender = 1	0.169	(−0.597, 0.972)
Propofol Infusion	−0.013	(−0.024, −0.002)
Propofol Bolus	−0.161	(−0.423, 0.105)
Midazolam	−5.937	(−26.201, 13.879)
Fentanyl	0.761	(0.136, 1.398)
Epilepsy_comorbid = 1	0.885	(−1.375, 3.189)
Group * Age	0.004	(−0.026, 0.034)
Group * Age 2	0.000	(−0.000, 0.000)
Group * Age 3	0.000	(−0.000, 0.000)
Sigma2	11.293	(9.662, 13.221)

*HPD, highest posterior density*.

However, there were notable differences in alpha power in ASD and NT patients at specific ages. At age 65 months (5.42 years), the mean alpha power was increased in NT (18.86 ± 0.68 dB) compared to ASD patients (17.65 ± 0.89 dB) with 56.5% certainty (Figure 3B). At age 270 months (22.5 years), the mean alpha power was increased in NT (11.89 ± 1.77 dB) compared to ASD patients (6.37 ± 2.50 dB) with 80.2% certainty (Figure 3B).

### The Trajectory of Slow (0.1–1 Hz) Power by Age Differs in ASD and NT Patients

**Figure 4:**
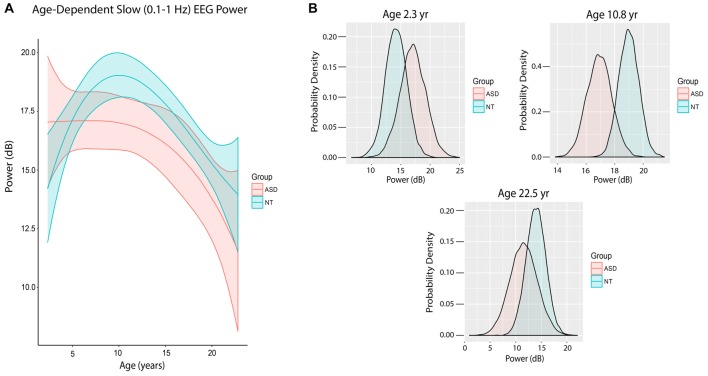
Slow (0.1–1 Hz) EEG power evolves differently with age in ASD patients.** (A)** Slow (0.1–1 Hz) power in the propofol-induced frontal EEG by age and ASD status. ASD patients at mean age experienced a significant reduction in slow power relative to NT patients. **(B)** Posterior distributions of slow (0.1–1 Hz) EEG power (dB) in ASD vs. NT cohorts at 2.3 years, 10.8 years and 22.5 years. Mean slow power was increased in ASD patients at 2.3 years, but decreased at 10.8 and 22.5 years.

Figure 4A shows the trajectory of slow power by age in ASD and NT pediatric patients after accounting for baseline characteristics between the cohorts, ASD patients at mean age experienced a significant 1.93 dB reduction in slow power compared to NT patients (80% credibility interval −3.38 to −0.54; Table 3).

**Table 3:** Posterior probabilities from primary model of slow (0.1–1 Hz) power (dB), using mean age.

Parameter	50%	80% HPD
Group = ASD	−1.926	(−3.382, −0.538)
Age	−0.024	(−0.041, −0.008)
Age 2	0.000	(−0.000, −0.000)
Age 3	0.000	(−0.000, 0.000)
Gender = 1	−0.428	(−1.255, 0.437)
Propofol Infusion	0.017	(0.006, 0.029)
Propofol Bolus	0.699	(0.386, 0.960)
Midazolam	36.503	(14.701, 57.802)
Fentanyl	−1.366	(−2.036, −0.684)
Epilepsy_comorbid = 1	3.246	(0.817, 5.729)
Group * Age	0.009	(−0.024, 0.041)
Group * Age 2	0.000	(−0.000, 0.000)
Group * Age 3	0.000	(−0.000, 0.000)
Sigma2	13.093	(11.1202, 15.328)

*HPD, highest posterior density*.

There were also notable differences in slow power in ASD and NT patients at specific ages. At age 28 months (2.33 years), the mean slow power was increased in ASD (17.05 ± 2.20 dB) compared to NT patients (14.20 ± 1.83 dB) with 52.5% certainty (Figure 4B). At age 130 months (10.8 years), the mean slow power was increased in NT (18.99 ± 0.70 dB) compared to ASD patients (16.93 ± 0.87 dB) with 80.8% certainty (Figure 4B). At age 270 months (22.5 years), the mean slow power was increased in NT (13.95 ± 1.91 dB) compared to ASD patients (11.56 ± 2.69 dB) with 41.3% certainty (Figure 4B).

### Increased Incidence of Burst Suppression in ASD and NT Patients

**Figure 5:**
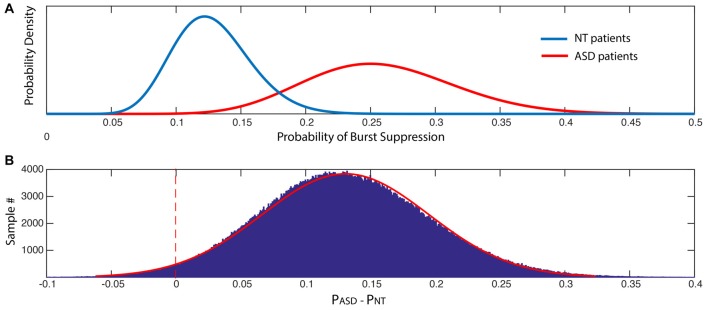
The probability of suppression events is significantly increased in ASD patients. **(A)** Posterior distributions of the probability of burst suppression in ASD and NT cohorts. **(B)** Histogram of the difference between the probability of burst suppression in ASD and NT cohorts, where P_ASD_ > P_NT_. The probability that P_ASD_ > P_NT_ was 0.9730.

ASD patients experienced an episode of burst suppression nearly twice as often as NT patients (23% vs. 12.2%, *p* < 0.05). The probability that ASD patients had greater incidence of burst suppression was 0.9730 (Figure 5).

We investigated the clinical characteristics of all cases with confirmed burst suppression to explore the potential causes of this finding (Table 4). The mean age of ASD patients was moderately higher than NT patients (17.48 vs. 14.67 years, SMD = 0.459), as was the rate of comorbid epilepsy (21.4% vs. 0%, SMD = 0.739). Interestingly, there was a similar male predominance in both ASD and NT cohorts (71.4% vs. 73.3%, SMD = 0.043). Compared to NT patients, ASD patients received reduced lower propofol infusion dose (2.59 vs. 3.26 mg/kg, SMD = 0.281) and propofol bolus dose (1.34 vs. 2.01 mg/kg, SMD = 0.424), but increased midazolam (0.01 vs. 0.00 mg/kg, SMD = 0.561) prior to the onset of suppression. There were no substantial differences in fentanyl dose prior to onset of suppression in ASD vs. NT patients (0.57 vs. 0.70 mg/kg, SMD = 0.141).

**Table 4:** Baseline characteristics and anesthetic regimen in ASD and NT patients who experienced burst suppression or prolonged suppression.

Parameter	ASD (*n* = 14)	NT (*n* = 15)	SMD
Age (months)	209.71 (66.50)	176.93 (76.16)	0.459
Male sex (%)	10 (71.4)	11 (73.3)	0.043
Propofol infusion (mg/kg) prior to suppression	2.59 (2.23)	3.26 (2.58)	0.281
Propofol bolus (mg/kg) prior to suppression	1.34 (1.77)	2.01 (1.38)	0.424
Midazolam (mg/kg) prior to suppression	0.01 (0.03)	0.00 (0.00)	0.561
Fentanyl (mcg/kg) prior to suppression	0.57 (1.21)	0.70 (0.57)	0.141
Comorbid clinically diagnosed epilepsy (%)	3 (21.4)	0 (0.00)	0.739

*All values are mean (SD). SD, standard deviation; SMD, standardized mean difference*.

## Discussion

In this study, we found age-dependent changes in EEG power during propofol-induced consciousness in ASD compared to NT patients. These changes may reflect underlying differences in the development of inhibitory GABAergic circuits in ASD. The propofol-induced EEG provides a unique, non-invasive method of measuring the brain’s response to a specific neuro-pharmacological probe acting at GABAergic sites that are thought to be central to the etiology of ASD. Our results also suggest how anesthetic management could be improved in ASD patients, given the high incidence of burst suppression observed in the ASD cohort in our study.

### Changes in the Age-Dependent Propofol-Induced EEG in ASD

The EEG measures scalp electrical potentials generated by cortical post-synaptic currents (Buzsáki et al., 2012). In adults, propofol induces stereotyped EEG oscillations that reflect the functional disruption of cortical and thalamocortical circuits. Propofol-induced slow oscillations reflect alternating “up” and “down” states in cortical neurons whose firing is silenced by periods of hyperpolarization, which may be mediated by networks of parvalbumin-positive (PV+) and somatostatin-positive (SS+) inhibitory neurons (Purdon et al., 2013; Funk et al., 2017; Zucca et al., 2017). Propofol-induced frontal alpha oscillations are thought to arise from amplification of inhibitory GABAergic signaling within cortical and thalamocortical circuits (McCarthy et al., 2008; Ching et al., 2010; Baker et al., 2014; Flores et al., 2017). Computational modeling studies have shown that putative PV+ fast-spiking interneurons may interact with pyramidal neurons to generate gamma oscillations (Börgers et al., 2005) that are crucial for sensory and cognitive processing (Cho et al., 2015) and large-scale network integration (Uhlhaas and Singer, 2010; Kitzbichler et al., 2015; Vakorin et al., 2017). Under propofol and other GABAergic anesthetics, this interneuron and pyramidal neuron circuit is slowed down by enhanced inhibition, causing the network to oscillate at alpha frequencies (McCarthy et al., 2008; Ching et al., 2010). In NT children, the propofol-induced EEG shows a striking age dependence. The frontal alpha oscillations are absent in infants (Cornelissen et al., 2015), but develop within the first year of life (Akeju et al., 2015; Lee et al., 2017; Cornelissen et al., 2018). Both propofol-induced frontal alpha and slow oscillations increase in power through the first 6–8 years of life, decreasing subsequently through adolescence and adulthood (Akeju et al., 2015; Lee et al., 2017; Cornelissen et al., 2018). These age-related changes likely reflect the trajectory of postnatal neurodevelopment, particularly within frontal cortical and thalamocortical circuits (Akeju et al., 2015; Lee et al., 2017; Cornelissen et al., 2018).

Overall, the structure of the propofol-induced frontal EEG in ASD patients closely resembled that of NT patients, displaying prominent slow-delta and frontally coherent alpha oscillations. However, these characteristic oscillations evolved differently with age in ASD vs. NT patients. Slow oscillation power was initially higher in ASD compared to NT patients, but progressively declined with age. Frontal alpha power was initially lower in ASD compared to NT patients, and continued to decline with age.

The changes in the age-dependent propofol-induced frontal EEG observed in ASD patients could reflect underlying neuropathology associated with ASD. Post-mortem studies have shown excessive cortical synaptic density in very young children with ASD (Hutsler and Zhang, 2010; Tang et al., 2014). This higher cortical synaptic density could correspond to higher postsynaptic currents and thus increased slow oscillation EEG power observed in young ASD patients in our study. Other studies have reported accelerated frontal cortical thinning in adolescent ASD patients, which could correspond to the progressive reduction in slow EEG power with age in our ASD cohort (Zielinski et al., 2014). ASD patients show reduced numbers of PV+ interneurons in medial prefrontal cortex, (Hashemi et al., 2017) reduced GABAergic signaling during sensory processing tasks, (Robertson et al., 2016) altered resting-state functional connectivity across multiple oscillatory bands including the gamma band, (Kitzbichler et al., 2015; Vakorin et al., 2017) and disruption of thalamocortical functional and anatomical connectivity (Nair et al., 2013). Given the putative fast-spiking PV+ interneuron-dependent mechanisms of the propofol-induced frontal alpha wave, encompassing both cortical and thalamocortical circuits, the progressive decline in alpha EEG power in ASD could correspond to pathology in these inhibitory circuits. Disruption of inhibitory networks could also lead to reduced slow EEG power in ASD patients, given recent studies suggesting that PV+ interneurons may participate in the generation of slow waves (Funk et al., 2017; Zucca et al., 2017).

### Increased Probability of Burst Suppression and Prolonged Suppression in ASD

General anesthetics that act primarily by enhancing GABAergic transmission, such as the halogenated ethers, propofol, and barbiturates, can induce burst suppression, a deep state of unconsciousness or coma beyond what is required for general anesthesia (Scheller et al., 1990; Van Ness, 1990; Rampil et al., 1991; Hartikainen et al., 1995a,b; Huotari et al., 2004). The mechanisms for burst suppression are poorly understood, but may involve neuronal metabolic changes leading to increased intracellular potassium driven by ATP-dependent K^+^ pumps, which can induce intermittent hyperpolarization and neuronal silence (Ching et al., 2012). Intracellular calcium dynamics may also play a role (Amzica, 2009). Intraoperative burst suppression becomes increasingly likely with age in adults, (Purdon et al., 2015a) and is an independent risk factor for postoperative delirium (Soehle et al., 2015; Fritz et al., 2016).

The typical developmental shift of GABA from an excitatory to inhibitory neurotransmitter depends on sequential maturation of the sodium-(potassium)-chloride co-transporter 1 (NKCC1) to potassium-chloride co-transporter 2 (KCC2; Lu et al., 1999; Rivera et al., 1999). ASD patients have been shown to have an increased NKCC1:KCC2 ratio, which tends to preserve excitatory GABAergic signaling with increased intracellular potassium and chloride in affected neurons (Lemonnier and Ben-Ari, 2010; Lemonnier et al., 2012; Duarte et al., 2013; Tyzio et al., 2014). Interestingly, administration of bumetanide, an NKCC1 inhibitor, has been shown to reduce ASD symptoms in both human patients and animal models of ASD, suggesting that these channels likely play a significant role in the changes in neurodevelopment leading to ASD symptomatology (Lemonnier and Ben-Ari, 2010; Lemonnier et al., 2012; Duarte et al., 2013; Tyzio et al., 2014).

Under the model of burst suppression proposed by Ching et al. (2012), an increased concentration of intracellular potassium would correspond to an increased propensity for burst suppression. Thus, the increased susceptibility to propofol-induced burst suppression we observed in ASD patients might reflect underlying differences in the NKCC1:KCC2 ratio that would lead to higher intracellular potassium in ASD patients compared to NT patients. Some ASD patients show decreased brain metabolism (Haznedar et al., 2006). This could also relate to burst suppression under the model in Ching et al. (2012), in that burst suppression may occur as a result of decreased brain metabolism.

Elderly adults receiving propofol for maintenance of general anesthesia show significantly reduced frontal alpha oscillation power and increased probability of burst suppression (Purdon et al., 2015a). We have observed these same effects in late adolescent ASD patients during propofol-induced unconsciousness, which may signify a form of accelerated neuronal aging in this patient population. As discussed earlier, adolescent ASD patients show accelerated cortical thinning and disruption of functional and structural connectivity, both of which are hallmarks of age-related neurodegeneration (McGinnis et al., 2011; Fjell et al., 2014).

### Limitations

This study has several potential limitations. First, medications were administered by clinical judgment of the anesthesiologist, and dosing was neither prospective nor controlled. However, after accounting for differences in medication administration in our modeling, we still found significant changes in age-dependent EEG power in ASD patients. This suggests that the differences we observed did not reflect differences in anesthetic management.

Our analysis revealed that there were moderate effect size differences of propofol infusion, propofol bolus, and midazolam dosing between the ASD and NT cohorts in both analyses. Based on our understanding of anesthesia-induced EEG changes in adult patients, reduced propofol dosing, as we saw in the ASD patients, would tend to diminish alpha and slow oscillations with elevation of beta/gamma (13–50 Hz) oscillations (Purdon et al., 2015b). We did not observe this in our data. In fact, we observed that autistic patients were more likely to be in burst suppression, which is associated with higher anesthetic doses. Benzodiazepines such as midazolam can also increase the likelihood of burst suppression (Wroblewski and Joseph, 1992). However, only a small subset of ASD patients in our study actually received midazolam premedication. Furthermore, the average dose was clinically insignificant at 0.01 mg/kg compared to the recommended dose of IV midazolam for preoperative anxiolysis of 0.05–0.10 mg/kg for pediatric patients (Miller et al., 2014). Such small doses of midazolam would not be expected to produce EEG changes of the magnitude we observed in our study.

Another limitation is that ASD is a highly heterogeneous disease, and the patients in this study represented a broad range of symptom severity and comorbidities. A subset of ASD patients also took psychoactive medications such as antiepileptics, antidepressants, and antipsychotics chronically, which could plausibly affect the EEG dynamics observed (Ouchi and Sugiyama, 2015). However, we believe that our ASD cohort is representative of the general ASD population presenting for anesthesia. In future work it would be interesting to characterize anesthesia-induced EEG oscillations as a function of ASD severity and co-morbidities. Nonetheless, the present study is representative of the general pathophysiology of ASD and suggests differences in clinical management that could be applicable to a broad population of ASD patients.

In the burst-suppression analysis, the mean age of ASD patients was higher than NT patients as was the frequency of comorbid epilepsy. Both of these characteristics could contribute to an increased probability of experiencing suppression events. However, it again seems unlikely that the magnitude of difference in incidence of suppression events between ASD and NT patients could be attributed to these qualities alone. For instance, in our previous studies of burst suppression in adults, the propensity for burst suppression increased with age, but required approximately two decades of aging to reach the 2-fold differences we observed here in young ASD patients (Purdon et al., 2015a).

## Conclusions and Future Directions

Here, we present evidence that ASD patients respond differently to the GABAergic drug propofol compared to NT patients, including reduced alpha EEG power with age and increased susceptibility to EEG burst suppression. Our results may reflect a form of neurodegeneration in cortical and thalamocortical inhibitory circuitry in ASD, as well as a possible difference in the ratio NKCC1:KCC2 receptors that would influence the propensity for burst suppression. Our studies also illustrate how GABAergic anesthetic drugs such as propofol can be used to characterize GABAergic neural oscillations and circuits in development and developmental disorders. Future directions include prospective, controlled-dose experiments that would minimize the potential confounding effects of variable anesthetic dosing that we needed to adjust for in this study. Future studies could also entail the collection of data regarding covariates such as genetic profile, level of intellectual disability, language proficiency and other parameters that are often used in stratifying ASD severity. This could make it possible to relate features of GABA-dependent anesthesia-induced oscillations in ASD patients to specific behavioral or cognitive deficits, or to specific genetic mutations.

## Author Contributions

EW, PF, ES, TB and PP designed the research. EW, JL, KT, and DZ performed data collection. EW, SB, TH, EB and PP performed data analysis. EW and PP wrote the article. All authors provided critical revision of the manuscript.

## Conflict of Interest Statement

The authors declare that the research was conducted in the absence of any commercial or financial relationships that could be construed as a potential conflict of interest.

## Supplementary Material

The Supplementary Material for this article can be found online at: https://www.frontiersin.org/articles/10.3389/fnsys.2018. 00023/full#supplementary-material
