# Supplementary figures and images for: Age-Dependent Changes in the Propofol-Induced Electroencephalogram in Children With Autism Spectrum Disorder
Source: Front Syst Neurosci. 2018 Jun 22;12:23. doi: 10.3389/fnsys.2018.00023 (PMC6024139; doi:10.3389/fnsys.2018.00023)

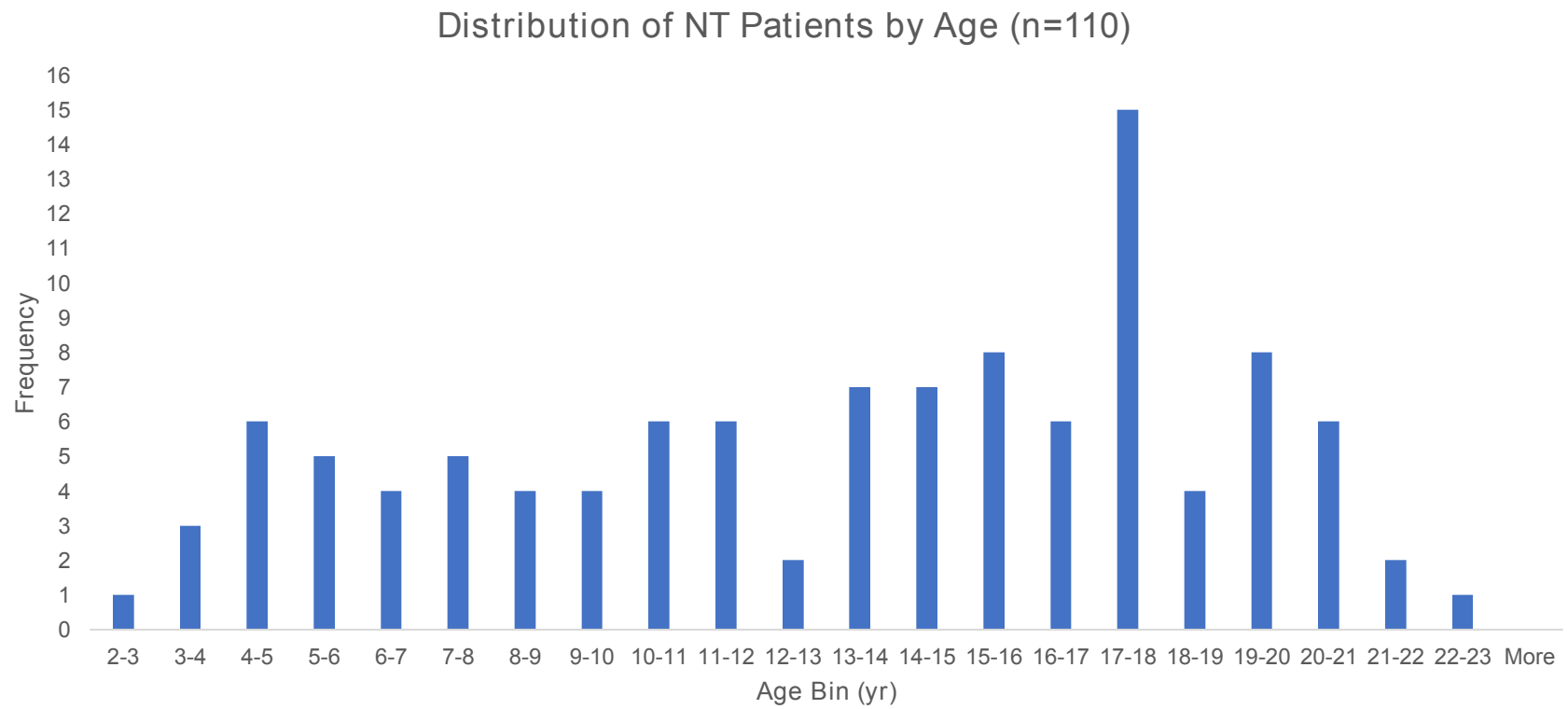

Figure S1a

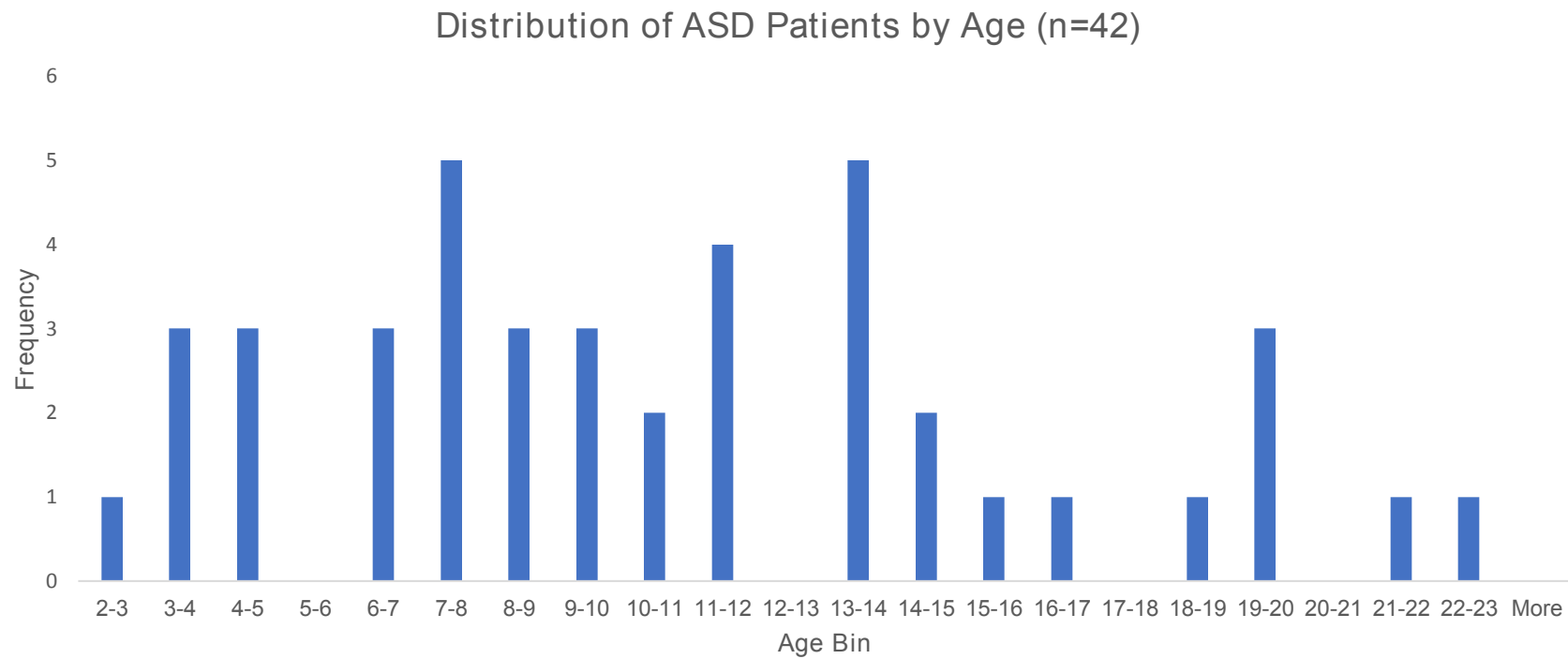

Figure S1b

Supplement: Supplementary file 1 [file Image_1.pdf]
